# A semantic strength and neural correlates in developmental dyslexia

**DOI:** 10.3389/fpsyg.2024.1405425

**Published:** 2025-02-04

**Authors:** Sladjana Lukic, Fei Jiang, Maria Luisa Mandelli, Ting Qi, Sarah M. Inkelis, Emily Rosenthal, Zachary Miller, Emma Wellman, Silvia A. Bunge, Maria Luisa Gorno-Tempini, Christa Watson Pereira

**Affiliations:** ^1^Memory and Aging Center, University of California, San Francisco, San Francisco, CA, United States; ^2^UCSF-UCB Schwab Dyslexia and Cognitive Diversity Center, San Francisco, CA, United States; ^3^School of Communication Science and Disorders, Florida State University College of Communication and Information, Tallahassee, FL, United States; ^4^Department of Epidemiology & Biostatistics, University of California, San Francisco, San Francisco, CA, United States; ^5^Department of Brain Cognition and Intelligent Medicine, Beijing University of Posts and Telecommunications, Beijing, China; ^6^Department of Psychology, University of California, Berkeley, Berkeley, CA, United States; ^7^Helen Wills Neuroscience Institute, University of California, Berkeley, Berkeley, CA, United States

**Keywords:** developmental dyslexia, semantic fluency, clustering and switching approach, cognitive strength, brain dynamics

## Abstract

**Introduction:**

Most studies of dyslexia focus on domains of impairment (e.g., reading and phonology, among others), but few examine possible strengths. In the present study, we investigated semantic fluency as a cognitive strength in English-speaking children with dyslexia aged 8–13.

**Methods:**

Ninety-seven children with dyslexia completed tests of letter and semantic verbal fluency, standardized measures of reading and cognitive functions, and task-free resting-state functional magnetic resonance imaging (rs-fMRI). First, we adjusted performance on semantic fluency by letter fluency and created a residual score that was used to separate participants into high (residual >0) or average (residual <0) semantic performance groups. We then employed a psycholinguistic clustering and switching approach to the semantic fluency task and performed dynamic task-free rs-fMRI connectivity analysis to reveal group differences in brain dynamics.

**Results:**

High and average semantic fluency groups were well-matched on demographics and letter fluency but differed on their psycholinguistic patterns on the semantic fluency task. The high semantic fluency group, compared to the average semantic fluency group, produced a higher number of words within each cluster, a higher max cluster size, and a higher number of switches. Differential dynamic rs-fMRI connectivity (shorter average dwell time and greater brain state switches) was observed between the high and average groups in a large-scale bilateral frontal-temporal-occipital network.

**Discussion:**

These data demonstrate that a subgroup of children with dyslexia perform above average on semantic fluency tasks and their performance is strongly linked to distinct psycholinguistic patterns and differences in a task-free resting-state brain network, which includes regions previously implicated in semantic processing. This work highlights that inter-individual differences should be taken into account in dyslexia and reveals a cognitive area of strength for some children with dyslexia that could be leveraged for reading interventions.

## 1 Introduction

Dyslexia is a neurodevelopmental disorder characterized by impairments in learning to read and/or spell (International Dyslexia Association, Lyon et al., 2003). Much research on dyslexia has focused on deficits in phonological processing (Griffiths and Snowling, 2001; Pennington, 2006; Vellutino et al., 2004), processing the basic sounds of words. Although great progress has been made using this focus, individual differences and other cognitive processes such as *semantic processing* have been less researched in dyslexia. Existing computational models of reading emphasize that reading is a byproduct of dynamic interactions of orthographic, phonological, and semantic processing systems. For instance, the parallel distributed processing model (Seidenberg and McClelland, 1989) emphasizes the importance of the dynamic product of these systems. The dual route cascaded model of reading aloud (Coltheart et al., 2001) characterizes three routes to reading: the non-lexical reading route (via grapheme-to-phoneme rule system), lexical non-semantic route (via orthographic/phonological input lexicons), and the lexical semantic route (via semantic system).

Semantics refer to the general knowledge of concepts and/or word meanings that can be empirically measured by receptive tasks, such as one-word picture matching tests, or expressive tasks, such as a semantic fluency test (Reilly et al., 2024). Several studies have examined performance on the semantic fluency test (timed verbal production of different words within a given category, e.g., animals) in English-speaking children with dyslexia. The findings were mixed: one study reported superior semantic fluency abilities (Griffiths, 1991), another found abilities similar to typically developing children (Frith et al., 1995), while a third found reduced abilities compared to typically developing children (Levin, 1990). Other cross-linguistic studies of semantic fluency across languages also provided inconsistent results, either showing no differences (Deacon et al., 2016; Smith-Spark et al., 2017), lower scores (Mengisidou et al., 2020), or higher utilization of semantic information (Rasamimanana et al., 2020; van der Kleij et al., 2019) in children with dyslexia compared to age-matched typically developing children. A semantic route to reading could be of particular importance for children with dyslexia as semantics, unlike phonology, may not be impaired (Everatt et al., 2008; see Deacon et al., 2016 for a review), and may even represent a strength in dyslexia (van Rijthoven et al., 2018; van Rijthoven et al., 2021). Furthermore, many of the studies on atypical development utilized small sample sizes and primarily reported total fluency, measured by the number of words produced per minute. However, a recent systematic review (Arán Filippetti et al., 2023) emphasizes the importance of analyzing psycholinguistic metrics by utilizing a fine-grained approach to measure clustering and switching during fluency tasks (Troyer, 2000; Koren et al., 2005). According to this review, 18 (out of 22) studies focused on clustering and switching strategies in individuals with atypical development, while only three studies specifically included children with dyslexia and/or developmental disorders (Mengisidou and Marshall, 2019; Mengisidou, 2020; Mielnik et al., 2015). Additionally, the performance of children with dyslexia on non-verbal design fluency tasks has been rarely investigated with verbal fluency tasks. This research could help determine whether deficits are primarily due to executive control issues rather than phonological processing difficulties, as highlighted by Smith-Spark et al. (2017). Their study compared phonemic, semantic, and design fluency tasks in adults with and without dyslexia, revealing that while phonological processing problems are central to dyslexia-related fluency deficits, executive control challenges cannot be entirely dismissed.

A few studies have also examined brain correlates of semantics in typically developing children using functional magnetic resonance imaging (fMRI). Of the existing studies, most converge on brain regions that have also shown similar neural activity in adults, namely the left inferior and middle frontal gyri and the middle temporal gyrus (Gaillard et al., 2003; Porter et al., 2011; Gonzalez et al., 2021). Recently, it has been suggested that children may also engage right hemisphere analogues regions during the performance of fluency tasks such as semantic fluency (Gonzalez et al., 2021). Other studies have mapped language functional connectivity in typical development (Friederici et al., 2011; Qi et al., 2021), however, task-free resting-state functional connectivity networks in children with dyslexia are yet to be defined (see Battistella et al., 2020 for “semantic” resting-state networks in adults). Task-free resting-state functional connectivity is a promising tool for brain research on dyslexia (Bailey et al., 2018; Finn et al., 2014; Schurz et al., 2015) because it can allow researchers to correlate behavioral differences with connectivity changes in the absence of task demands and find underexplored areas of strength. Specifically, dynamic task-free rs-fMRI connectivity is a new framework for understanding brain function that can provide the dynamic changes that may underly many behaviors and has been used across several diseases (Yang et al., 2020). The time-varying dynamic network analysis method proposed by Yang et al. detects micro brain state changes over time during rest. These microstates are not directly observable in practice. The method allows for the calculation of mean dwell time, which represents the average duration a subject remains in a particular state before transitioning to another. A longer dwell time suggests that brain states change less frequently, potentially reflecting less efficient brain processing (Jin et al., 2023).

In this study, we aimed to test specific psycholinguistic contributors to semantic fluency performance in a subset of English-speaking children with dyslexia and task-free brain dynamics that may differentiate good performers. Our objectives were to elucidate psycholinguistic patterns related to individual semantic retrieval ability using clustering and switching approaches and examine underlying brain differences that may relate to a semantic strength using a task-free resting-state fMRI. Our ultimate goal is to understand how children with dyslexia think and learn in the context of neurodevelopmental differences and leverage their cognitive strengths to provide strength-based learning alternatives to the standard deficit-based model. We expected that the children who demonstrated a strength on the semantic fluency test (independent of phonology) would use a different psycholinguistic strategy to complete fluency tasks (see Arán Filippetti et al., 2023 for a review of empirical studies highlighting the importance of utilizing qualitative analysis of fluency task output). We also expected that these children would show greater efficiency in the semantic control network – such as the left inferior and middle frontal gyri and temporal regions (see Enge et al., 2021 for meta-analysis of fMRI studies on semantics)–during a task-free resting state, compared to children who did not demonstrate this strength.

## 2 Method

### 2.1 Participants

A total of 97 children with dyslexia participated in the study, selected from a larger sample of children recruited from the database of the Dyslexia Center at UCSF (see inclusion/exclusion criteria below), and were part of multidisciplinary research program that performs neurological, psychiatric, cognitive and language evaluations of children with Developmental Dyslexia (DD). Participants underwent comprehensive academic, cognitive and language assessments, and were given a diagnosis of DD based on International Dyslexia Association (IDA; Lyon et al., 2003; *Definition Consensus Project*) definition. All met inclusion criteria: (1) native speakers of English language, (2) 8–13 years of age, (3) intelligence estimates within normal limits (WASI Matrix Reasoning or Receptive One-Word Picture Vocabulary >5th percentile; Wechsler, 1999), and (4) adequate sensorimotor capacity, including normal or corrected-to-normal vision and hearing. Reading ability was measured by using standardized age-adjusted measures. A participant was excluded from the dyslexia cohort if all their reading scores fell above the 25^th^ percentile: isolated timed word and pseudoword reading (TOWRE-2 Sight Word Efficiency and Phonemic Decoding Efficiency; Torgesen et al., 2012), untimed word and pseudoword reading (Woodcock Johnson Letter Word Identification and Word Attack; Schrank et al., 2014), and/or paragraph reading (GORT measures; Wiederholt and Bryant, 2012). All children were screened from schools in Northern California.

### 2.2 Materials and procedure

#### 2.2.1 Semantic fluency test

We used the Word Generation test from the second edition of “A Developmental Neuropsychological Assessment,” Second Edition (NEPSY-II) to probe individual differences in semantic ability. All participants completed the semantic fluency test where they named as many items as they could from a given category (animals then food/drink) in a limited amount of time (60 s). Because this test is timed, we sought to account for speed of verbal processing. Therefore, all participants also completed (1) the letter fluency test from the NEPSY-II, where participants report all the words they can think of that start with a particular letter (F then S), regardless of the letter sound, in 60 s and (2) the design fluency, where participants produce unique drawn figures that fit specified criteria. The advantages of these fluency tests are that they do not require a participant to read or give a written response and they are quick to administer (one-minute tests).

**Figure 1:**
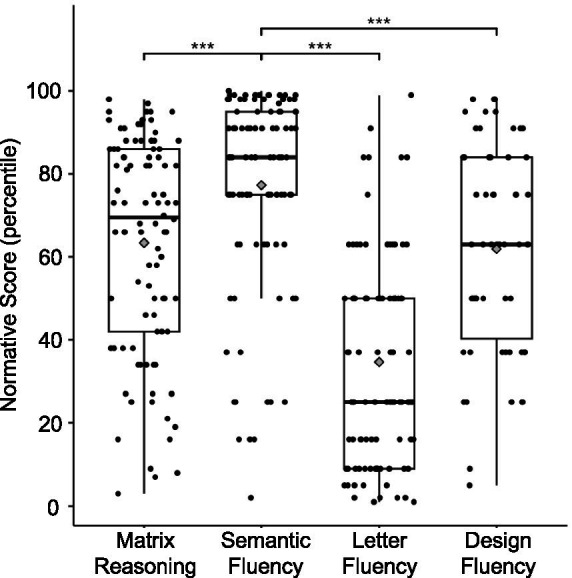
Task performance (normative percentile scores) for the children with dyslexia. Performance on semantic fluency was significantly higher compared to performance on matrix reasoning, letter fluency, and design fluency tests, *p* < 0.001 (***). Center lines show the medians, gray diamonds show the means, and box limits indicate the 25th and 75th percentiles of the sample.

In general, participants with dyslexia performed much higher on semantic fluency test compared to letter fluency or design fluency tests (see Figure 1). To isolate semantic abilities in dyslexia, we created residual scores out of semantic fluency percentiles by adjusting for letter fluency percentiles (to help control for influences related to phonology skills and domain-general and verbal processing speed) in a cohort of children with dyslexia. Accordingly, the participants were subdivided into two groups based on their semantic fluency residual scores: average (<0; *N* = 34) and high semantic fluency (>0; *N* = 63). See Supplementary Figure S1 for Semantic Fluency residuals frequency distribution histogram. Demographics, reading scores, and significant group differences are provided in Table 1.

**Table 1:** Demographic variables and reading test performance for the high and average semantic fluency groups.

	Semantic fluency group		
	High	Average	
	*N* = 63	*N* = 34	*P/V*
Demographics			
Gender			0.975
Female	28 (44.4%)	15 (44.1%)	
Male	35 (55.5%)	19 (55.8%)	
Handedness			0.355
Right-handed	59 (93.7%)	30 (88.2%)	
Non-right-handed	4 (6.3%)	4 (11.8%)	
Age at assessment (years)	10.2 (1.4)	10.4 (1.4)	0.668
Matrix Reasoning (median (25, 75))	68.5 (42, 86)	71.5 (38, 86)	0.996
Receptive One-word Picture Vocabulary Test	79 (47, 91)	69 (54, 83)	0.473
Family Income: median (range)	$300 k–$399 k ($80 k–$500 k+)	$300 k–$399 k ($100 k–$500 k+)	0.589
ADHD	22 (34.9%)	15 (44.1%)	0.374
Fluency tests			
Semantic fluency *	91 (84, 98)	63 (25, 75)	<0.001/0.793
Letter fluency	25 (9, 50)	31 (9, 50)	0.565
Reading			
Sight word efficiency (timed real word)	5 (1, 19)	7.5 (2,18)	0.592
Phonemic decoding efficiency (timed pseudoword)	5.5 (2,19)	6 (2,19)	0.812
WJ letter word identification (untimed real word)	19 (6, 32)	19 (9, 34)	0.768
WJ word attack (untimed pseudoword)	25 (14, 47)	30 (19, 50)	0.383
GORT comprehension	16 (9, 37)	16 (5, 37)	0.532

Number of participants, means and standard deviations for demographics and medians (25th, 75th) or range (for ordinal data) are reported, as well as *p-value/Cramer’s V* effect sizes of group differences. * Test used to divide participants into high and average groups. Percentiles reported unless otherwise specified.

Additionally, the semantic fluency test lends itself to quantitative and qualitative analyses of semantic knowledge. A systematic review by Arán Filippetti et al. (2023) highlights the importance of analyzing psycholinguistic metrics, including (1) the number of clusters (a subset of words that are associated by a theme and produced contiguously; e.g. pig, cow, horse as farm animals under the set of animals), (2) the max cluster size (the number of words in the largest cluster; e.g., pig, cow, horse, zebra, giraffe would demonstrate max size of 2), and the number of switches (the number of times a subject switches from one cluster to another; e.g. pig, cow, horse, zebra, giraffe would demonstrate one switch between horse and zebra from farm animals to African Savanna animals). Therefore, we utilized a fine-grained clustering and switching approach (Troyer, 2000; Koren et al., 2005) to analyze if an individual had higher number of segments of connected semantic retrieval (i.e., larger number of clusters) and/or employed more cognitive switches (i.e., larger number of switching between clusters) during the semantic fluency task.

#### 2.2.2 Standardized tests

Participants also completed two standardized clinical tests to assess cognitive abilities, such as verbal short-term memory measured by CVLT C Trial 1 (Fridlund and Delis, 1994), and cognitive flexibility measured by Children’s Colored Trails B (Williams et al., 1995). In the CVLT C Trial 1 test, the participants were asked to immediately recall a list of words after being read a word list that contains three different categories of items, which taps into verbal short-term memory. The Children’s Colored Trails B test requires participants to draw lines that simultaneously sequence numbers and switch between two colors, which measures cognitive switching. We included these two high-order cognitive measures to examine strength in executive function measures along with semantic strength. This would allow us to explore between-group differences.

### 2.3 Neuroimaging

#### 2.3.1 rs-fMRI data acquisition

Participants underwent a research MRI protocol within 6 months of their neuropsychological evaluation. Neuroimaging data were acquired at the Sandler Neuroscience Center at UCSF. Participants with a 3.0 Tesla Siemens Prisma MR scanner equipped with a 64-channel head coil were included in the neuroimaging analysis (*N* = 68, 70%).

The protocol consists of a T1-weighted 3D magnetization prepared rapid acquisition gradient echo (MPRAGE) acquired with 160 sagittal slices, TE/TR/TI = 2.98/2300/900 ms, flip angle = 9°, isotropic voxel with size of 1 mm, field-of-view = 256 × 256 mm^2^, matrix = 256 × 256, iPAT = 2 and a task-free resting-state functional MRI (rs-fMRI) acquired with a single-shot echo planar imaging. Three volumes consisting in 68 AC/PC-aligned axial slices acquired in interleaved order with the following parameters: TR/TE = 1290/32 ms, multiband factor of 4; flip angle = 45°, slice thickness = 2.2 mm, in-plane resolution = 2.2 × 2.2 mm; field-of-view = 211 mm.

To minimize head movement during rs-fMRI data acquisition, participants were instructed to remain still with their eyes closed, and cushions were used to stabilize their heads. Moreover, given that head motion tends to be high in children populations, we implemented a strategy wherein participants were given a 20-min break outside of the scanner during the session. This method has been demonstrated to effectively mitigate head motion in functional MRI studies in children (Meissneret al., 2020). Following one of the breaks, the rs-fMRI acquisition lasting 6 min was conducted.

#### 2.3.2 rs-fMRI pre-processing

rs-fMRI data were analyzes using in-house pipeline that employed tools from FSL v5^1^, AFNI^2^, and Numpy (Python v2.7.3). To establish T1 equilibrium, the first five volumes of the acquisition were discarded. Subsequently, the rs-fMRI images were corrected for slice timing and checked for excessive motion. Five participants were excluded for excessive motion (criteria were: a maximum of 2 mm for relative head motion, a maximum of 2° for relative rotation, and a maximum of 10% of the total frames with motion spikes, calculated as relative motion >1 mm) (*N* = 63, 65%). The mean functional image of each participant was then registered with the 3D MPRAGE using a rigid body transformation.

Next, the structural image was normalized to the MNI space, and the transformation matrix was applied to the rs-fMRI data, which were finally spatially smoothed using a Gaussian kernel with a full width at half maximum (FWHM) of 6 mm. Cerebrospinal fluid (CSF) and white matter (WM) tissue probability maps, calculated with the FSL FEAT tool, were then used to compute the mean time-series used as regressors. Subsequently, the functional data underwent bandpass filtering (0.008 Hz < *f* < 0.15 Hz), and nuisance variables were regressed out, including the six motion parameters, CSF and WM time series, as well as their first derivative and quadratic terms, as suggested by Satterthwaite et al. (2013). The combination of spectral filtering and nuisance regression was performed as a single step using AFNI tools (Hallquist et al., 2013).

### 2.4 Analyses

#### 2.4.1 Quantitative and qualitative analysis of semantic fluency test

The total number of correct words produced (excluding errors and repetitions) was computed for each participant. For example, according to the NEPSY-II scoring procedure (Brooks et al., 2009), one point is given for each correct response (e.g., “grizzly bear” or “cat” for animals, and “beef” or “water” for food/drinks), then correct responses for category 1 (animals) and category 2 (food/drinks) are summed for *the semantic fluency total scores*. Established and easily recognizable mythological creatures (e.g., “unicorn”) are considered correct responses and are given one point. Similarly, distinct dishes (e.g., “cashew-chicken”) are considered correct responses and are given one point, rather than two points for each component in the item. Repetitions, nonsense words, non-category words, and proper nouns are not considered correct responses and are not awarded points.

To examine children’s abilities to create and/or shift between semantic subcategories on the semantic fluency test, we performed a clustering and switching approach analysis. Subcategories of animals and food/drinks were determined *a priori* (see Appendix). This classification scheme was based on those described in previous studies: 9 animals subcategories using Crowe and Prescott (2003), and 9 food/drinks subcategories using Troyer (2000). Animals were classified based on their typical habitat/environmental context (e.g., farm/domestic, wild/zoo, aquatic animals, etc.) and taxonomic relations (Crowe and Prescott, 2003) rather than on their living environment and human use (Troyer et al., 1997). Food/drinks clusters were classified based on the naturally-occurring items (e.g., fruits, vegetables, dairy products, meats, etc.; Troyer, 2000). Each word was assigned to one of the 9 subcategories by three independent coders (EW, VYHP, AF). The coders were instructed to code an item that could appear in the two clusters according to immediately preceding or subsequent words (e.g., avocado was coded either as a fruit or vegetable depending on the words around it: fruit if preceded by apple and vegetable if preceded by potato; similarly, penguin was coded either as a bird or wild/zoo animal depending on the context provided by neighboring words).

Next, the three variables were derived from each semantic category for each participant: (1) *Sum cluster size* (the total number of words that are embedded within a cluster), (2) *Maximum (max) cluster size* (the number of words in the largest cluster), and (3) *Number of switches* (one less than the number of clusters).

Given that no standardized method of analysis of cluster formation for this version of semantic fluency existed, we adopted scoring rules for clustering and switching defined by Koren et al. (2005). According to this approach, clusters are defined as two or more related words produced consecutively rather than single words clusters. Therefore, switches were calculated as the number of transitions between clusters excluding single words. Thus, the number of switches is the number of clusters minus one.

Given that each fluency variable is dependent on the number of words produced (total fluency), two different positions have been taken in the past literature: some researchers computed a ratio by dividing each score by the total number of words produced (Raskin et al., 1992), while others claim that using ratio scores would be equivalent to correcting a cause for its effect (Koren et al., 2005; Troyer, 2000). In the current study, we report both raw values and ratios for numbers of switches and sum cluster size; we also report maximum cluster size, because this metric is not dependent on the total number of words produced.

#### 2.4.2 Statistical analyses of behavioral data

Means and standard deviations were calculated for all fluency variables for each category (animals, food/drinks) and executive function measures separately for each semantic fluency group (average, high). Given that the patterns were similar in animal and food/drinks categories (see Supplementary Table S2), we combined them for the one-way analysis of variance (ANOVA) between the groups on demographic and psycholinguistic measures. As aforementioned, the groups were determined by a residual semantic fluency score after controlling for letter fluency; the high group had residuals >0, the average group had residuals <0. Cognitive measures percentile scores were analyzed using Kruskal-Wallis where the semantic fluency group was the independent variable.

#### 2.4.3 rs-fMRI data analyses

A novel dynamic rs-fMRI connectivity analysis was employed (Yang et al., 2020) to investigate underlying brain networks subserving semantic strength in children with dyslexia. We selected dynamic rs-fMRI connectivity analysis for two main reasons. First, it enhances our sensitivity to temporal changes in network states. Second, it is well-suited to our study, which focuses on the neurocognitive contributions, particularly from language and executive function systems, required for rapid word retrieval and the inhibition of inappropriate word choices within a semantic category (Betzel et al., 2016; Shine et al., 2015; Zalesky et al., 2014).

The pre-processed rs-fMRI data were analyzed using a time-varying dynamic network approach (Jiang et al., 2022; Jin et al., 2023), a technique that measures changes in brain network connections over time. It has two components: a temporal component (given by the number of state transitions and their dwell times), which describes how brain states change over time (dynamic), and a spatial component (given by the number of eigenmodes), which represents the static connections between brain regions. The model separates these components and uses piece-wise constant multivariate signal generation described in detail in Jiang et al. (2022) and Jin et al. (2023). The brain regions were anatomically localized using the Brainnetome Atlas (https://atlas.brainnetome.org; Fan et al., 2016). We followed the method of Jin et al. (2023) and extracted the spatial features of whole-brain rs-fMRI connectivity (all 246 brain regions of interest). We applied time-varying dynamic network analysis to data from each participant, generating spatial results where the model produces a 246-length spatial feature vector, with each value corresponding to a region of interest (ROI). To assess differences between the two groups, we performed a t-test on each of the 246 spatial features. We then identified the brain regions that had significantly different spatial features between the high and average semantic fluency groups. To account for multiple comparisons, we apply Bonferroni correction, considering an ROI significant if the *p*-value from the t-test is less than 0.05/246. Thus, all results were peak-level and cluster-level corrected (*p*_FWE_ < 0.05) for multiple comparisons in the whole-brain analysis.

## 3 Results

### 3.1 Performances on standardized cognitive tests in two semantic fluency groups

On average, percentile performance across fluency tests ranged from 2 to 99.99 percentiles, with mean percentile performance of 77.3 (SD = 23.85) on semantic fluency and 34.67 (SD = 24.85) on letter fluency across groups. The median age-normed percentile and interquartile range for the fluency tests are displayed in Table 1, separately for each group. The two semantic groups were matched on letter fluency, matrix reasoning, and vocabulary.

Both high and average groups were highly accurate on CVLT-C Trial 1, with a mean percent correct accuracy of 60.8 ± 27.8 and 45.6 ± 30.2, respectively. However, the high and average groups were less accurate on the Children’s Colored Trails B test, with a mean percent correct accuracy of 28.3 ± 20.4 and 28.3 ± 18.9, respectively. We found a significant main effect of group on CVLT-C Trial 1 [*F*_(1,94)_ = 6.193, *p* = 0.014, Cohen’s *f* = 0.26] and not on Children’s Colored Trails B [*F*_(1,91)_ = 2.292, *p* = 0.133, Cohen’s *f* = 0.16], but this did not survive correction for multiple comparisons.

### 3.2 Clustering and switching patterns on semantic fluency in two semantic fluency groups

We had item-by-item data on the semantic fluency test for 77 of the initial 97 participants (see Supplementary Table S1 on 53 children in high and 24 children in average semantic fluency group); these participants’ data were included in clustering and switching analyses. This subgroup did not differ meaningfully from the full sample on demographic variables. See Supplementary Table S1 on statistics of demographics between the two groups selected for clustering and switching analyses.

#### 3.2.1 Inter-rater reliability

Three coders independently coded each response on semantic fluency tests into one of 9 subcategories of animals or food/drinks. The coders were three research assistants trained by the first author (SL). Inter-rater reliability was calculated in R using percentage agreement and Cohen’s Kappas. On semantic animal fluency, inter-rated reliability was %-agree = 93.8, Kappa = 0.92 for rater 1 vs. rater 2, and %-agree = 95.7, Kappa = 0.94 for rater 1 vs. rater 3. On semantic food/drinks fluency, inter-rated reliability was %-agree = 91.2, Kappa = 0.89 for rater 1 vs. rater 2, and %-agree = 93.8, Kappa = 0.93 for rater 1 vs. 3. All correlations were significant at the 0.001 level and given that Cohen’s kappa falls in the 0.8–0.9 range (near-perfect agreement), we adopted the coding from rater 1 in our analyses below.

#### 3.2.2 Semantic fluency variables

Given that similar results were observed across the two semantic categories (animals, food/drinks) (see Supplementary Table S2) on fluency variables, the results reported below included sums across the two semantic categories to yield sum and maximum cluster size scores and the total number of switches in the two semantic fluency groups. Participants generated an average of 35 (SD = 8.7) total correct words. High and average semantic fluency groups produced an average of 38 (SD = 7.2) and 27 (SD = 5.3) total correct words, respectively (see Table 2).

**Table 2:** Semantic fluency variables for the two semantic fluency groups.

	Sum cluster size	Sum cluster size ratio (out of # words)	Max cluster size	# switches (# clusters − 1)	# switches ratio (out of # words)
High (*n* = 53)	18.94 (5.51)	0.48 (0.09)	3.76 (1.31)	7.58 (2.52)	0.19 (0.05)
	4–30	0.20–0.68	2–7	1–14	0.05–0.29
Average (*n* = 24)	11.42 (3.71)	0.42 (0.10)	2.79 (1.20)	4.83 (1.93)	0.18 (0.05)
	4–18	0.22–0.59	1–6	2–9	0.10–0.26
*F* (1, 75)	37.05	6.32	9.62	22.54	1.85
Sig.	**<0.001**	**0.01**	**0.003**	**<0.001**	0.18
Mean Sq	935.86	0.06	15.62	125.07	0.01
Residuals	25.26	0.01	1.62	5.549	0.003
Cohen’s *f*	0.70	0.29	0.36	0.55	0.16

Means (standard deviations), and *F*-statistics for group comparisons are reported.

**Figure 2:**
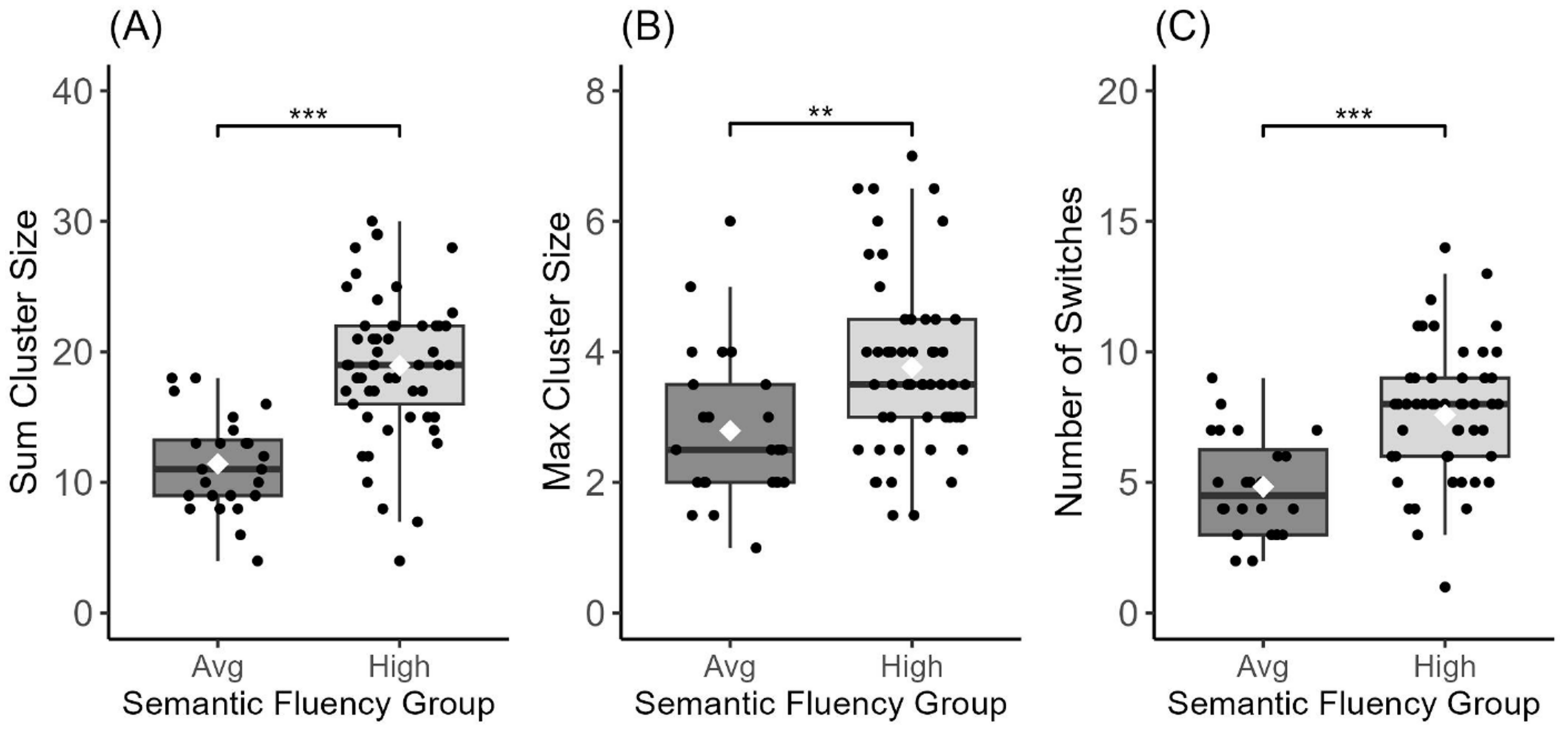
Semantic fluency variables for the high and average semantic fluency groups. Sum Cluster Size **(A)**, Max Cluster Size **(B)**, and Number of Switches **(C)** all showed a significant group difference; *** < 0.001, ** < 0.01. Center lines show the medians, white diamonds show the mean, and box limits indicate the 25th and 75th percentiles of the data.

In between-group analyses, we found a significant main effect of group on sum cluster size [*F*_(1,75)_ = 37.05, *p* < 0.001], max cluster size [*F*_(1,75)_ = 9.62, *p* = 0.003], and number of switches [*F*_(1,75)_ = 22.54, *p* < 0.001]. *Post-hoc* analyses via Tukey’s HSD test revealed higher sum cluster size and max cluster size as well as a higher number of switches for the high semantic group relative to the average semantic group on all three fluency variables (all *p*-values <0.05; see Figure 2 and Table 2). These group differences persisted after using a ratio (by dividing each score by the total number of words produced) for sum cluster size [*F*_(1,75)_ = 6.323, *p* = 0.014] but not for number of switches [*F*_(1,75)_ = 1.851, *p* = 0.177; Table 2].

### 3.3 Differential dynamic resting-state functional connectivity in two semantic fluency groups

**Figure 3:**
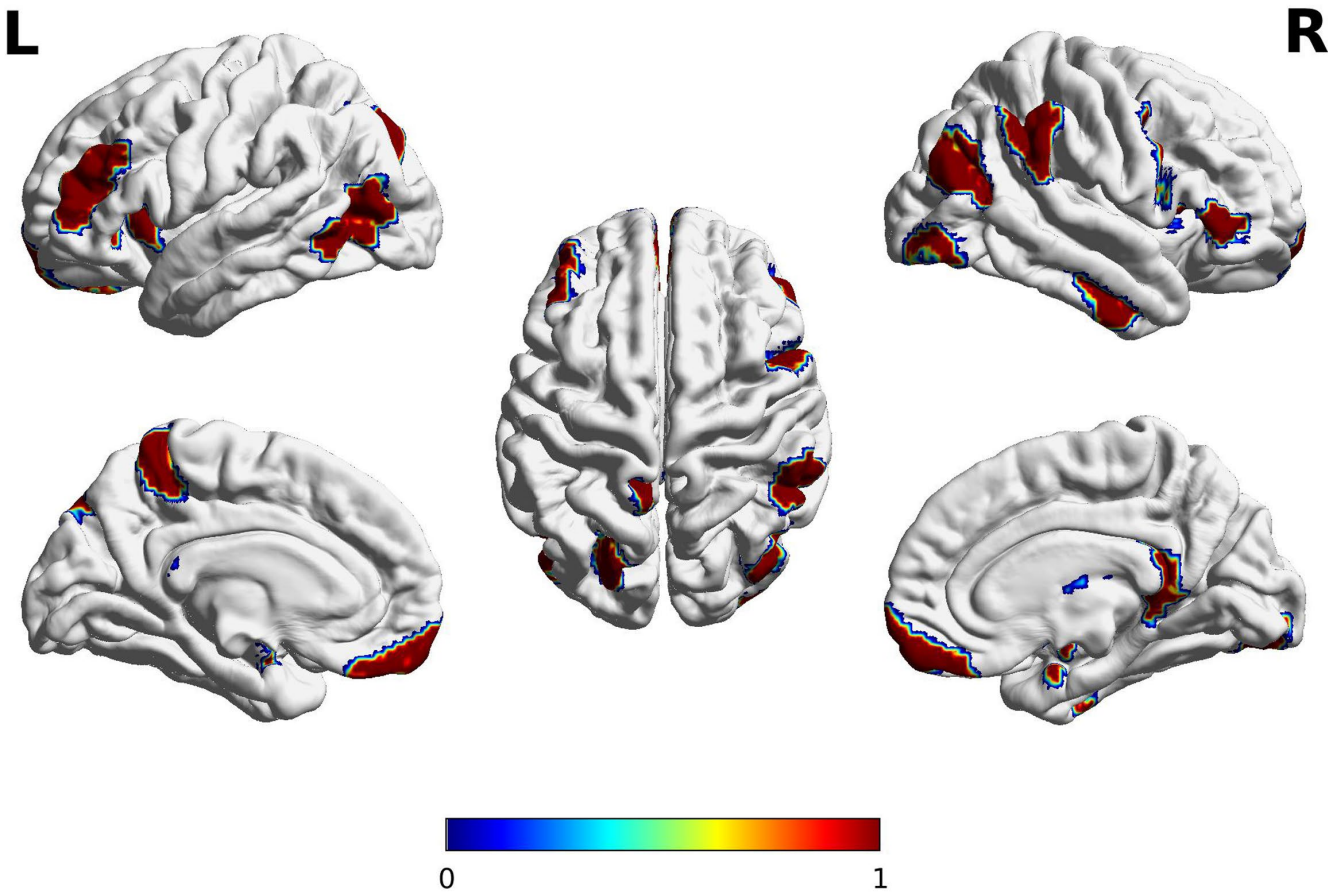
Brain regions emerging from a dynamic task-free resting-state functional connectivity analysis in two semantic fluency groups. The red color bar reflects significant regions where the high semantic group is greater in the variations of spatial features compared to the average semantic group. Significant group differences in spatial features (average of brain states over time) were observed in the left inferior and middle frontal gyri (IFG/MFG), inferior temporal gyri (ITG), and dorsal medial parietal lobe as well as right IFG, ITG, inferior parietal, temporoparietal junction. The clusters extended subcortically to include bilateral basal ganglia and amygdala, and right hippocampus, and thalamus. The anatomical locations of significant clusters were confirmed using the Brainnetome atlas (https://atlas.brainnetome.org/bnatlas.html). The images were peak-level and cluster-level corrected (*p*_FWE_ *< 0*.05) for ROIs covering the whole brain. The spectrum of colors is due to the contouring smoothing that occurs during the plotting.

The high semantic fluency group demonstrated different internal states of the brain (i.e., spatial features from the dynamic functional connectivity) compared to the average semantic group (*p* < =0.05/246, where 246 is the number of ROIs) in the left inferior and middle frontal gyri (IFG/MFG), posterior inferior temporal gyri (ITG), and dorsal medial parietal lobe as well as right IFG, ITG, inferior parietal, temporoparietal junction, and occipital regions (see Figure 3). The results also revealed that the high semantic fluency group exhibits a significantly shorter average dwell time in this network of regions, suggesting that their brain states change more frequently compared to the average semantic fluency group. The high semantic fluency group switches more frequently between brain states compared to the average semantic fluency group do on average (number of change points: 7.4706 vs. 5.862, respectively; p-value = 0.037). This is consistent with the observation that the maximal dwell time in a stationary time segment is significantly shorter in the high semantic fluency group than the average semantic fluency group with (52.754 vs. 83.611, respectively; *p*-value = 0.055). These results suggest that the high semantic fluency group is significantly more active in brain state switches. The significant ROIs demonstrating the significant dynamic rs-fMRI connectivity are listed in Supplementary Table S3.

## 4 Discussion

Despite the central importance of semantics and domain-general abilities in child development and reading acquisition, these research lines have advanced in a segregated fashion. Too few studies have been conducted that examine whether these might be areas of strength for children with dyslexia. The goal of the present study was to determine individual differences in semantic skills (i.e., how children represent and process knowledge) in children with dyslexia, whether these relate to domain-general abilities, and whether behavioral differences are related to differences in functional brain networks. We identified two groups of children with dyslexia with distinct semantic fluency performance that show equal phonological impairment and are matched on demographic variables. The high semantic fluency group, compared to the average semantic fluency group, produced a higher number of words within each cluster (sum), a higher max cluster size, and a higher number of switches. Preliminary dynamic rs-fMRI connectivity analyses revealed group differences in brain dynamics, namely, the high semantic fluency group had more switches between brain states compared to the average semantic fluency group, indicating more efficient brain function. These differences were observed in a large-scale bilateral frontal-temporal-occipital network of regions, which has been previously implicated in semantic processing in other fMRI studies. These findings suggest developmental differences in brain networks and word retrieval strategies that might subserve semantic strengths in some children with dyslexia.

### 4.1 Greater semantic fluency performances in dyslexia

We believe the strong performance in semantic fluency in children with dyslexia is related to semantic adeptness but isolating semantics from other cognitive skills is challenging, similar to other studies (Aita et al., 2018). However, once we regressed letter fluency on semantic fluency and grouped children based on a residual score, we were able to obtain well-matched groups aside from semantic fluency performance. The particular semantic ability identified would likely be more related to the production or selection of specific words based on lexical-semantic knowledge rather than comprehension of higher-level semantics, such as deep cohesion or referential cohesion (e.g., Dahl et al., 2020). Similarly, semantic fluency categories are usually based on high-frequency, concrete categories that may not be representative of all aspects of semantics, particularly abstract or lower-frequency categories. For example, it is common for children to participate in everyday conversations about animals or food/drink, but it is less common for them to participate in conversations about evolution or non-poisonous plant species (Williams et al., 2013). Therefore, this strength in semantics should be interpreted within the confines of the types of categories used, i.e., concrete, high frequency, and the means of examination, i.e., production.

While interpreting results, it is important to consider whether children with dyslexia have a strength in semantics or are using semantics to compensate for their difficulties with phonology. First, ironically, part of that debate is a matter of semantics in the sense that compensation compared to alternate route needs to be better defined in order to frame the debate. Two key areas to describe are the order of development and the typical distribution of work. If one ability is acquired first and strength in the other ability is only noted when the first ability is compromised, then compensation may be an appropriate term. On the other hand, if development is simultaneous and strength is normally distributed, then alternate is likely a better description. Similarly, if a particular ability requires multiple cognitive skills and there is a normal distribution of a balance of skills, then alternate would be a better term to characterize strengths. Finally, most of the studies suggesting children with dyslexia use semantics to compensate for difficulties with phonology are specifically based on reading (Levesque et al., 2017). Our semantic fluency results demonstrate that within dyslexia there is a distribution of semantic ability that is independent of phonology. Further, semantic fluency does not differentiate reading ability within our sample of youth with dyslexia, because our groups are matched on those abilities. Therefore, we favor the interpretation that, in our data, semantic fluency serves as a strength rather than a form of compensation.

Several studies have examined semantic fluency in English-speaking children with dyslexia. The study by Levin (1990) reported that children with dyslexia had lower verbal fluency, including semantic fluency, compared to age-matched controls. Levin assessed fluency in a way similar to our study (number of correct words per minute) and used a similar category (food). However, there are a few important caveats to Levin’s study: (1) they reported that the dyslexia group generated 14.1 names compared to 10.4 names generated by the control group in their Table, while the statistics reported in the text claimed the opposite. Additionally, the mean number of words generated for controls does not sum within a small rounding error even if the proper names are reversed between dyslexia and controls, which suggests there were likely data sorting errors. Thus, the actual results of the study are in question; (2) The groups were not clearly matched on general intelligence, and they did not report means and standard deviations on the measure used for an intelligence proxy (Peabody Picture Vocabulary Test), which associates with fluency tests (Friesen et al., 2021; Regard et al., 1982), and (3) only male participants were included, which may influence performance on certain fluency tests (Hirnstein et al., 2023).

A second study found that children with dyslexia had stronger semantic fluency performance, average design fluency, and lower phonemic fluency compared with controls (Griffiths, 1991). This pattern of findings is similar to the distributions of semantic, design, and letter fluency performances in our data, and the age and intelligence scores of their participants were similar to our cohort. However, results cannot be directly compared, as they used semantic cueing methods of extracting fluency, i.e., saying a word that corresponds to a cue within a certain time interval (cue: big; response: elephant) and replacing a nonsense word with a real word based on a series of five descriptive sentences. Additionally, their participants were English-speaking but Scottish, which may influence education approaches and/or vocabulary. Lastly, a third study (Frith et al., 1995) also used a different method from ours to interrogate semantic fluency and recorded how many seconds it took for participants to say 10 correct words. They found that the children with dyslexia were slower than controls to produce words based on phonemic but not semantic cues. Their participants were also older than our participants by roughly 2 years. However, despite these differences, a similar pattern was found in our study in that the children with dyslexia had difficulties with phonemic but not semantic fluency.

Notably, the children in our study primarily attended private schools that are designed to teach children with learning difficulties. Therefore, it is possible that the higher normed percentiles score we obtained on semantic fluency reflects the impact private schools have on students’ semantics rather than higher than average innate abilities in children with dyslexia (Jacobsen et al., 2017). Although our study was not designed to directly address this potential confound, there are a few variables in our dataset that provide some insights. First, the semantic fluency normative scores within our cohort are higher than vocabulary, nonverbal reasoning, and other fluency measures, which indicates relative strength in semantics within the cohort. Second, not all children in our cohort performed above average on the semantic fluency task (i.e., members of the average semantic fluency group), but they went to the same schools and had similar vocabulary, nonverbal reasoning, and family income compared to high semantic fluency participants. These ancillary observations may suggest the strength we identified cannot be entirely accounted for by private schooling.

Lastly, according to past research, semantic cognition can be broken down into two components: semantic control and semantic knowledge (Lambon Ralph et al., 2017; Vonk et al., 2020). On the one hand, semantic strength might be related to the ability to retrieve more words from memory within the semantic network (*semantic knowledge for a given subcategory*) and hold them in verbal short-term memory. On the other, semantic strength might be associated with higher cognitive flexibility (*cognitive control processes*) that support semantic memory retrieval. Our groups did not show differences in semantic knowledge as measured by the Receptive One-Word Picture Vocabulary Test. We did see some differences in executive functions subserving semantic retrieval, such as verbal short-term memory (CVLT-C Trial 1), but these differences did not withstand correction for multiple comparisons. The CVLT-C Trial 1 may be more likely than other verbal short-term memory measures (e.g., digit span) to associate with semantic cognition specifically because of its embedded semantic categories. It is possible that if someone implicitly detects the embedded structure, they may also perform better on the task than someone who does not. This is in line with other studies showing relationships between verbal fluency and short-term and working memory (Aita et al., 2018; Marques et al., 2022), but these studies were confounded by phonological impairment. The semantic cognition components are particularly relevant to our findings on clustering and switching during the semantic fluency task (as discussed below). Some children with strong semantic abilities may use strategic processes that enable them to generate words within a subcategory by leveraging their capacity to access more words from semantic memory. Additionally, they may efficiently shift to a new subcategory when the current one is exhausted, relying on semantic control processes.

### 4.2 Psycholinguistic correlates of semantic strength in dyslexia

To further examine the psycholinguistic correlates of the semantic fluency task, we employed a clustering and switching analysis. Traditionally, clustering and switching analysis have been used to detect subtle differences in cognitive processes across the lifespan, in healthy aging (Amunts et al., 2020; Gonzalez-Burgos et al., 2019) and in various clinical disorders including neurodegenerative diseases (van den Berg et al., 2022; Wright et al., 2023), bipolar disease (Weiner et al., 2019), schizophrenia (Barattieri di San Pietro et al., 2023), traumatic brain injury (Woods et al., 2016), and stroke (Carpenter et al., 2021). Fluency tests was found to be associated with other cognitive abilities such as creative processes (Ovando-Tellez et al., 2022). Few studies have used this analysis in children (Hurks et al., 2010; Kavé et al., 2008) and even fewer have examined this in children with dyslexia and/or developmental language disorder (see Arán Filippetti et al., 2023 for review). Although controversies exist regarding the methods of quantifying a semantic search strategy (Voorspoels et al., 2014), we chose a widely used and studied approach (Troyer, 2000).

The clustering and switching analysis revealed that the high semantic fluency group outperformed the average semantic fluency group on raw clustering and switching variables, clustering ratio, and max cluster size, which is not impacted by the total number of words generated. They produced more words overall, had a higher number of clusters and switches, and produced larger clusters of semantically related items at their max performance. The groups did not differ on the ratio of switching/total number of words produced, which suggests that, in general, both groups of children switched between clusters at a similar rate, roughly every five words.

More research is needed to determine the pattern of performance in clustering and switching components in child clinical populations. Based on a recent systematic review (Arán Filippetti et al., 2023), out of the 33 studies that were reviewed, 18 analyzed clustering and switching strategies in participants with atypical development and only three included children with dyslexia and/or developmental disorders (Mengisidou and Marshall, 2019; Mengisidou, 2020; Mielnik et al., 2015). Accordingly, different profiles are observed in atypical development, with patterns that distinguish between impairment in cognitive flexibility (less switching) and the organization of semantic and phonological representations (smaller cluster size). Similarly, our findings suggest that children with semantic strength might implement a strategic process that helps them to generate words within a subcategory (larger cluster size and higher number of clusters) and to shift efficiently to a new subcategory when a subcategory is exhausted (higher number of switches). These findings may suggest developmental changes in the structure of children’s semantic networks (Vales et al., 20,220) which may shape controlled semantic retrieval (Marko and Riečanský, 2021).

### 4.3 Neural correlates of semantic strength in dyslexia

Dynamic rs-fMRI connectivity has been used to study task-free resting-state in several disease states (Yang et al., 2020). Similarly, we used dynamic rs-MRI to examine a semantic strength in dyslexia, and whether group differences were apparent in the brain’s spontaneous dynamics and spatiotemporal patterns. The results revealed that the high semantic fluency group exhibited a significantly shorter average dwell time, implying that these individuals may remain in a single brain state for shorter periods and may switch between brain states more efficiently. This study is model-based and exploratory, aimed at identifying potential explanations for group differences. Since microstates are not directly observable by humans, there is no definitive “ground truth” for the time spent in each state. The purpose of this analysis was to identify meaningful and interpretable features that could provide insights into underlying brain processes.

The dynamic rs-fMRI connectivity analysis specifically revealed a large-scale brain network potentially supporting semantic strength, including the left IFG/MFG, posterior ITG and medial parietal lobe, and the right IFG, inferior parietal regions, and occipitotemporal regions. This brain finding at rest is supported by the functional neuroimaging studies in healthy adults (see meta-analyses by Wagner et al., 2014) and lesion studies in stroke patients (Biesbroek et al., 2021) which have shown that word search during category-fluency tasks rely on the coordinated activity of several brain areas including the left IFG/MFG and posterior regions of the MTG and ITG as well as the parahippocampal and fusiform gyri. The finding is also in line with studies showing better fluency performance associated with larger surface area in brain regions related to semantic fluency in typically developing children (Gonzalez et al., 2021), and those showing specialization of executive systems (Satterthwaite et al., 2013) and semantics (Wang et al., 2021) during brain development, and is consistent with recent meta-analyses of semantic cognition in children (Enge et al., 2021). The data might suggest that higher connectivity of the so-called “semantic network” facilitates efficient information flow associated with easier switching and bigger cluster size during semantic fluency.

A key strength of our well-matched participant groups is that they were recruited from the same schools, received evidence-based reading interventions, and have similar reading scores. As a result, it is unlikely that the differences in brain dynamics are due to differences in exposure to reading instruction. Instead, it is more plausible that the differences in performance on the semantic fluency test and in the time-varying dynamic network reflect a characteristic, though potentially malleable, distinction between the semantic fluency groups. Some limitations of the study include unequal sample sizes for the high and average semantic fluency groups. It is also unfortunate that we do not have a control sample with similar socioeconomic metrics and no reading concerns to whom we could compare semantic fluency, psycholinguistic, and imaging results. Additionally, it will be important to examine semantic processing in students who are more representative of the population, i.e., diverse samples, particularly in terms of race, bilingualism, family income, and students enrolled in public schools. Finally, our imaging study was relatively small and preliminary, so larger studies that attempt to replicate the finding and also test for a continuous relationship with behavior will be valuable. Further out-of-sample validation is also required to confirm that the brain dynamics we have identified are generalizable across different studies. Given that our study was cross-sectional, it will also be important to conduct longitudinal studies that examine the change in brain dynamics before and after reading interventions. Future research would also benefit from investigating whether similar or different neurocognitive mechanisms are observed in both semantic and letter fluency tasks using the same dynamic task-free resting state approach and in both children with and without dyslexia, as this could provide deeper insights into their interplay within dyslexic populations.

## 5 Conclusion

The results of this study provide evidence of a semantic strength in dyslexia that is subserved by certain psycholinguistic strategies and task-free resting-state brain dynamics. These findings can inform future work in dyslexia by encouraging researchers to consider investigating strengths in dyslexia, cognitive approaches to tasks, and brain systems that may be used differently rather than deficiently. We hope this work will also inspire reading interventionists to consider a possible alternate route to reading that relies more heavily on a child’s strengths. Morphology is a linguistic element that has semantic information and can be taught systematically. Reading interventions in dyslexia that use morphology as a basis for reading may provide additional benefits after interventions in phonology have been applied.

## Data Availability

The datasets presented in this article are not readily available because the conditions of our ethics approval do not permit public archiving of anonymized study data. Data generated by the UCSF MAC are available upon request. Data requests can be submitted through the UCSF MAC Resource Request form: http://memory.ucsf.edu/resources/data. Access will be granted to named individuals in accordance with ethical procedures governing the reuse of sensitive data. All requests will undergo UCSF regulated procedure, thus requiring submission of a Material Transfer Agreement (MTA) which can be found at https://icd.ucsf.edu/material-transfer-and-data-agreements. No commercial use would be approved. Requests to access the datasets should be directed to Christa.Watson@ucsf.edu.

## Footnotes

^1^ http://fsl.fmrib.ox.ac.uk/fsl/fslwiki/

^2^ http://afni.nimh.nih.gov/afni/
